# Comparison of the Learning Pace of Medical Students and Surgical Residents in a Virtual Reality Laparoscopic Simulation

**DOI:** 10.7759/cureus.85855

**Published:** 2025-06-12

**Authors:** Anastasia Pikouli, Dimitrios Papakonstantinou, Evika Karamagioli, Emmanouil Pikoulis, Konstantinos Nastos, Dionysios Dellaportas, Nikolaos Pararas, Maria Gkova, Andreas Pikoulis, Charalampos Charalampous, Nikolaos Kaldis, Michail Thomaidis, Anastasios Giannou, Panagis M Lykoudis

**Affiliations:** 1 3rd Department of Surgery, University Hospital “Attikon”/National and Kapodistrian University of Athens, Athens, GRC; 2 Directorate of Epidemiological Surveillance and Intervention for Infectious Diseases, National Public Health Organization (EODY), Athens, GRC; 3 Department of General, Visceral, and Thoracic Surgery, University Medical Center Hamburg-Eppendorf, Hamburg, DEU; 4 Department of General Surgery, Liver, Pancreas, and Intestinal Transplantation, Hospital Universitario, Fundacion Favaloro, Buenos Aires, ARG; 5 Division of Surgery & Interventional Science, University College London (UCL), London, GBR

**Keywords:** laparoscopic surgery, learning pace, simulation, surgical training, virtual reality

## Abstract

Surgical training has evolved from the traditional Halstedian concept to include simulation, such as box trainers or virtual reality (VR), until a certain level of proficiency is achieved. This study aimed to compare the learning pace of medical students and surgical residents in laparoscopic simulation to assess the need for different training curricula. The research sample included 49 participants, consisting of 34 medical students and 15 surgical residents. Individual performances were recorded using the LAP Mentor III platform (Simbionix Corporation, Cleveland, OH, USA), and a learning curve analysis was conducted. No significant difference was observed between the two groups in terms of the learning pace. Additionally, there were no significant differences observed between students and residents in any parameter. A common curriculum in basic laparoscopic skills is suggested for both students and residents.

## Introduction

Surgical training has seen a substantial change over the past few years. The traditional Halstedian concept of “see one, do one, teach one” is now considered suboptimal, and the focus has shifted towards simulation training outside of the operating room (OR) until a certain level of proficiency has been reached [1, 2]. The main aspect of proficiency-based training is defining a benchmark for individual skills and procedures, which can be attained through structured training, following which performance is maintained at the desired level [2].

The simulators used in this proficiency-based training are mostly box trainers and virtual reality (VR) simulators. These platforms demonstrate promising results in improving skills and transferring them to real-life surgery when compared to standard training [3-6]. VR simulators offer the advantages of high flexibility, a wide range of different tasks, up to complete procedures, and direct performance measurement. To efficiently train students and residents, it is necessary to utilize various tasks and modalities and to arrange them in an appropriate sequence until proficiency in the desired skillset or procedure is achieved. Several curricula have been published, and their effectiveness has been supported by evidence, with the trainees being mostly first-year surgical residents or medical students. The performance of the aggregate of these two groups is compared to that of surgeons in order to evaluate construct validity [7-10]. However, whether there are differences between final-year medical students and novice surgical residents had not been addressed in a structured manner at the time of this study. This raises the question of whether these two groups have distinct performance characteristics that require different training curricula. As the goal (benchmarked performance scores) stays the same, certain aspects need to be examined. The first concerns optimal duration and number of repetitions needed for each group until the plateau component of the learning curve is achieved. The second aspect addresses the question of whether retention of skills differs between the two groups. The third aspect concerns the potential heterogeneity of learning curves for each of the desired skills.

The aim of this study was to compare the learning curves between medical students and surgical residents on a VR simulation curriculum of basic laparoscopic skills in order to assess the need for different training curricula.

## Materials and methods

The study was conducted in a single VR laparoscopic simulation center at the University General Hospital "Attikon", School of Medicine, National and Kapodistrian University of Athens in Athens, Greece, under the supervision of the university's 3rd Department of Surgery. Data recording occurred between December 2022 and July 2023. Inclusion criteria for participation as a trainee consisted of being either a surgical resident or an undergraduate medical student with no prior experience in VR laparoscopic simulation. Enrollment was on a voluntary basis, following invitations through the university’s communication platforms. 

The induction of recruited trainees was carried out by an experienced tutor in groups of up to six trainees. During the first encounter with the simulator, a brief overview of the history of simulation, its medical applications, and details concerning the simulator were provided. Familiarization with tasks lasted for approximately 30 minutes. The LAP Mentor III platform was used (Simbionix Corporation, Cleveland, OH, USA), operating on the Laparoscopic Basic Skills Module software, version 2.0.1.82 (Medical Simulation Corporation (MSC), Salt Lake City, UT, USA). The frequency and duration were predetermined, whereby each trainee engaged in a daily 20-minute session for at least three days per week. 

Tasks description 

The first task was about the manipulation of a 0° endoscope. Trainees were asked to identify nine balls sequentially, approach them with the scope until a virtual frame visually confirmed appropriate focus, and then click the sole button of the camera to take a virtual snapshot of the ball. Each ball would remain active for 20 seconds, and if it was not photographed within this timeframe, it would disappear, leading to the next one. Following that, a tenth ball would appear on screen, this time moving in a clockwise orbit, and trainees were asked to follow the ball while remaining in appropriate focus. 

The second task was about the manipulation of a 90° scope and was, in principle, similar to the previous one. The difference was that the first nine balls could be hidden behind virtual objects and crevices, and trainees would need to take advantage of the angled view in order to find them. 

For the third task, which was about eye-hand coordination, two instruments (hooks) were available, one marked with a blue ribbon and the other with a red one. Trainees had to insert the instruments, one at a time, through the trocars until they were visible on the screen. As soon as instruments appeared, three balls at different areas of the virtual space would appear. The first one would start blinking, indicating it must be touched with the tip of an instrument, and the color of the instrument should match the color of the ball. Once the ball was touched within 15 seconds, the next one would start blinking. If the ball was not touched within this timeframe, it would turn gray, and the next one would start blinking. The task included a total of three sets of three balls each. 

The aim of this fourth task was the placement of clips on flexible ducts, through which water flowed in a pool. Trainees had to insert two clip applicators. Water flow through the ducts would occur at random intervals. The medial segment of each duct was color-marked green. Trainees had to apply a clip on the green segment of each duct while water was flowing through it. The task ended when all nine ducts (arranged in three sets of three ducts each) were clipped or when the water level went above a red line close to the surface of the pool. 

The fifth task was similar to the previous one but also required the use of both hands in a complementary manner to safely grasp, pull, and clip leaking ducts. The trainees had at their disposal one grasper and one clip applicator. Unlike the previous task, the medial segment of the ducts was marked red. Trainees had to grab a tube close to each end, stretch it until the red segment became green, and then apply a clip on the green segment. As in the previous task, clipping was only possible while the duct was leaking and only within the green segment. The task ended when all nine ducts were clipped or when the water level reached the red line. 

The sixth task was about coordination on two-handed maneuvers. The task was composed of three sets, each one involving three red balls covered by a gel and a basket. Trainees had to move the gel away from the balls so that they turned green, and then sequentially put them in the basket. The task would end when the last ball was either placed in the basket or dropped outside it. 

In the seventh task, trainees had one grasper and one pair of straight scissors. The virtual prompt was composed of a circular frame attached to the floor of the virtual environment and a jelly-like, disc-shaped material attached to the frame by numerous virtual strings. Trainees had to grasp the jelly-like material, stretch it until adhesions were clearly visible, and subsequently cut them in a circular fashion. The task ended when all adhesions were divided. 

The eighth task was about the efficient use of diathermy, and trainees had one hook diathermy on each hand, activated by the corresponding foot pedal. The virtual environment included two vertical poles, on which several blue strings were transversely attached at various angles. One string would appear green, and trainees had to use hook diathermy to divide it. Following the division of the green string, another one would turn green until the last one was divided. The purpose of the task was to divide all strings using diathermy only when in contact with the appropriate string. Therefore, trainees had to use their instrument in order to hook the green string away from adjacent blue strings and cease pressing the foot pedal as soon as possible after the division of the string. 

The ninth task was composed of three-dimensional objects (two low-profile cylinders, two four-faced pyramids, and two cubes) with a different color on each of their faces. Each object appeared along with a “ghost” object, indicating the exact position the first one should be placed at. Using two graspers, trainees had to lift the object and rotate it by translocating it between the instruments until it looked like the ghost and position it at the appropriate place. Once done, the object and corresponding “ghost” would disappear, and the next set of objects would appear. The task ended upon the appropriate positioning of the sixth object.

Metrics’ thresholds 

For camera manipulation at 0°, success was defined by maintaining a horizontal view for at least 80% of the overall duration of the task. For the second task, accurate photo shooting was required, defined as hitting the button not more than once while not in appropriate focus. For the third task, trainees had to avoid touching non-blinking balls, as well as touching balls with a non-corresponding color-wise instrument or with any other part of it apart from its hook tip. Pass on the fourth task was achieved if the ducts were clipped in time, without misplacing more than one clip. Pass on the fifth task was achieved if the ducts were clipped in time, without misplacing any clip, and with less than 70 movements in total. The sixth task required correctly positioning all nine balls in their corresponding baskets with fewer than 50 movements. The seventh task required no more than 20 cutting maneuvers. The electrocautery task required at least 85% efficiency, measured as the time electrocautery was used while the hook was in contact with the appropriate band over the total time electrocautery was used. Lastly, in the translocation task, trainees had to complete the task with no more than 30 translocations. 

Thresholds were extrapolated and modified from the initial study that examined them [7]. 

In order to provide an element of a stepwise approach, tasks were arranged in three sets. The first set was comprised of Tasks 1-7. The second set consisted of Tasks 5, 6, and 8, and the third set consisted of Tasks 6, 8, and 9. Trainees had to achieve two consecutive full pass runs in one set before moving to the next one. The curriculum was considered completed when two consecutive full pass runs were achieved on the third set of tasks. 

Data collection and storage 

At the beginning of the curriculum, all trainees were asked to fill out a questionnaire that contained demographic data, year of studies/residence, present surgical residency, dominant hand, and previous experience with laparoscopic surgery, video games, and musical instruments (Appendix A). Performance data of each trainee was extracted directly from the simulator on a spreadsheet file (Microsoft Excel, Microsoft Corp., Redmond, WA, USA). Data were anonymized with the use of a serial number, and the correspondence between serial numbers and trainees was only known to the senior author. Every trainee had provided written consent for data recording, maintaining, processing, and publishing anonymously. 

Statistical analysis 

The statistical processing was conducted using the statistical package IBM SPSS Statistics software, version 25 (IBM Corp., Armonk, NY, USA). Descriptive statistics consisted of median and interquartile range for numerical variables and absolute number and class percentage for categorical variables. The normality of the data distribution was examined by visual analysis of frequency histograms and with the application of the Kolmogorov-Smirnov test. For continuous numerical data with a normal distribution, parametric tests (t-test, ANOVA, Spearman's correlation) were applied. For continuous numerical data with a non-normal distribution, non-parametric tests (Mann-Whitney U test, independent samples median test) were applied. For categorical data, χ² and Fisher’s exact test were used. The learning curves were analyzed using the serial comparison method, presented in a previous publication by the same senior author [8]. Two-tailed tests were applied wherever possible. P-values of <0.05 were considered statistically significant. Parameters that were analyzed are described in Table 1.

**Table 1:** A list of the parameter numbers and their corresponding tasks, along with a brief description of each parameter and its pass threshold. Parameter 1: Maintaining the horizontal view while using the 0° camera (%); Parameter 2: Accuracy rate—touched targets (%); Parameter 3: Accuracy rate—touched targets (%); Parameter 4: Number of clipped ducts; Parameter 5: Number of lost clips; Parameter 6: Total number of movements; Parameter 7: Number of clipped ducts; Parameter 8: Number of lost clips; Parameter 9: Total number of movements; Parameter 10: Number of lost balls that miss the basket; Parameter 11: Total number of cutting maneuvers; Parameter 12: Efficiency of cautery (%); Parameter 13: Number of translocations

Parameter	Associated task number	Description of the parameter	Pass threshold
1	Task 1: Camera manipulation 0^o^	Maintaining the horizontal view while using the 0° camera (%)	>= 80 %
2	Task 2: Camera manipulation 30^o^	Accuracy rate - touched targets (%)	>= 80 %
3	Task 3: Eye-hand coordination	Accuracy rate - touched targets (%)	>= 80 %
4	Task 4: Clip applying	Number of clipped ducts	>= 9 Clipped ducts
5	Task 4: Clip applying	Number of lost clips	<= 1 Lost clips
6	Task 5: Clipping and grasping	Total number of movements	<= 70 Movements
7	Task 5: Clipping and grasping	Number of clipped ducts	>= 9 Clipped ducts
8	Task 5: Clipping and grasping	Number of lost clips	<= 0 Lost clips
9	Task 6: Two-handed maneuvers	Total number of movements	<= 50 Movements
10	Task 6: Two-handed maneuvers	Number of lost balls that miss the basket	<= 0 Balls
11	Task 7: Cutting	Total number of cutting maneuvers	<= 20 Maneuvers
12	Task 8: Electrocautery	Efficiency of cautery (%)	>= 85 %
13	Task 9: Translocation of objects	Number of translocations	<= 30 Translocations

Post hoc power analysis was performed using an online calculator [11] that was accessed on the 30^th^ November 2023. The sample size for a two-sample Wilcoxon Mann-Whitney U-Test calculator was used (Version 1.060) [11]. The following options were selected: calculate power, calculate P(X>Y) from means and standard deviations assuming normal distributions, unequal sample sizes, Bonferroni correction, and alpha two-sided equal to 0.025. Mean values and standard deviations were inserted, and the two group sizes were specified. Relevant sample size calculation was performed on the same platform, selecting the sample size option and the following settings: calculate P(X>Y) from means and standard deviations assuming normal distributions, unequal sample sizes, Bonferroni correction, alpha two-sided equal to 0.025, and allocation ratio relevant to the study’s sample. 

## Results

**Figure 1:**
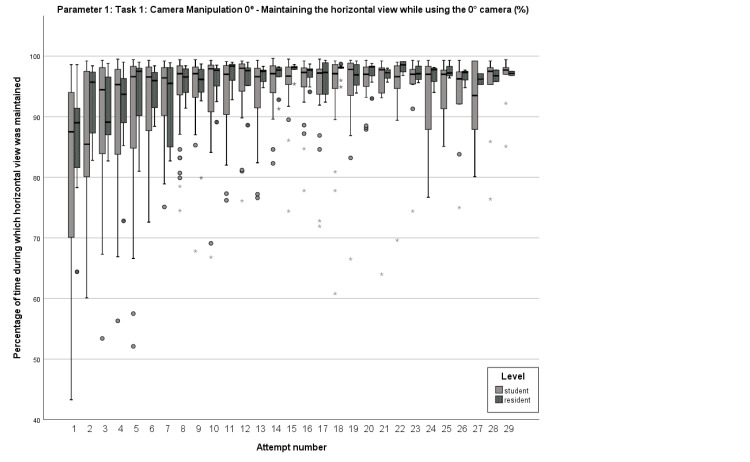
Box plot comparison of the learning curves regarding Parameter 1 Comparison of the percentage of time during which horizontal view was maintained while using the 0° camera in Task 1 between students and residents for each attempt.

The research sample included 49 participants, consisting of 34 medical students and 15 residents. Learning curves were clearly visualized for Parameter 1 (maintaining the horizontal view in Task 1), Parameter 6 (total number of movements in Task 5), Parameter 9 (total number of movements in Task 6), and Parameter 11 (total number of cutting maneuvers in Task 7). Parameter 1 displayed a median starting performance of 87.5% amongst medical students, with a first pass achieved on the initial attempt and a definitive pass reached on the second try. The respective median value for surgical residents was 89%, while the first and definitive passes were identical to those of the former group (Figure 1).

**Figure 2:**
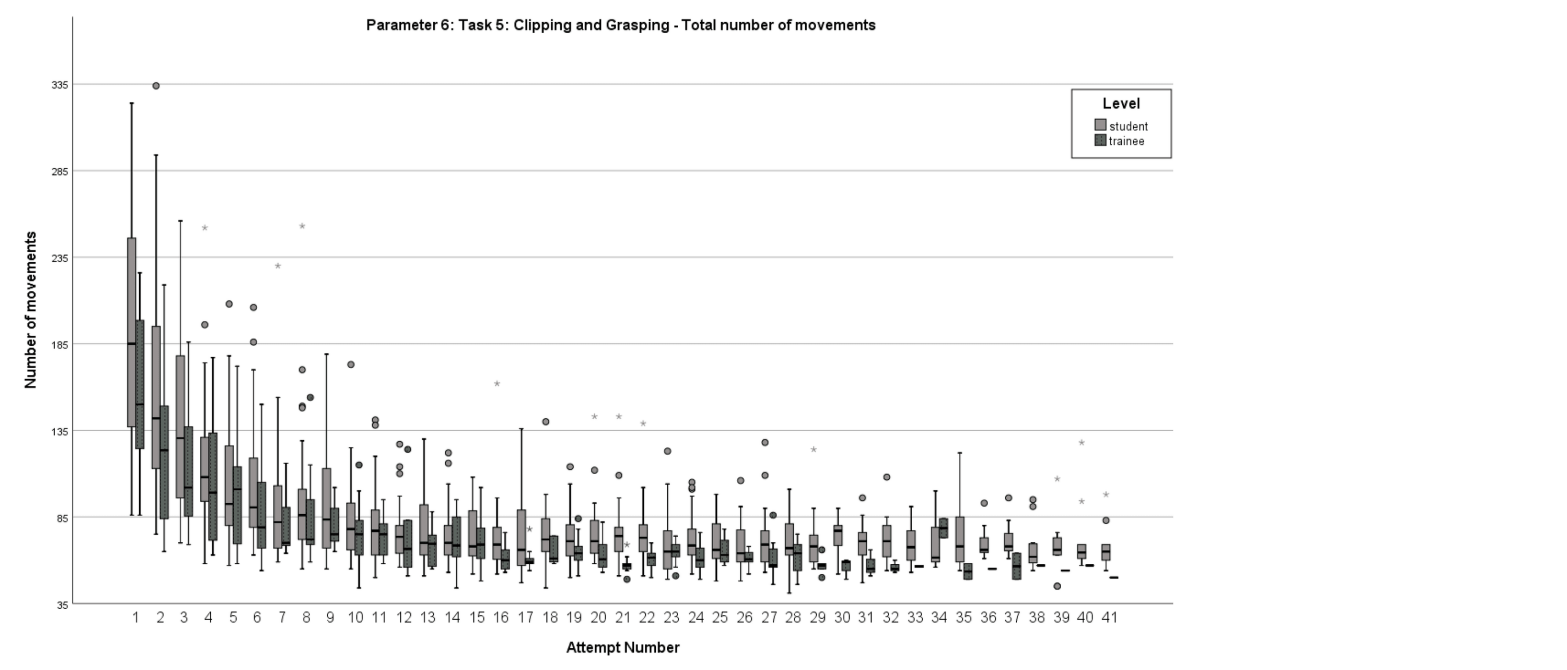
Box plot comparison of the learning curves regarding Parameter 6 Comparison of the total number of movements in Task 5 between students and residents for each attempt.

In terms of Parameter 6, students initially scored a median of 185 movements, with the first pass acquired on the ninth attempt and the definitive pass secured on the 14^th ^attempt. Conversely, the median number of movements for residents was 150, with the first pass obtained on the seventh attempt and the definitive pass attained on the 12^th^ attempt (Figure 2).

**Figure 3:**
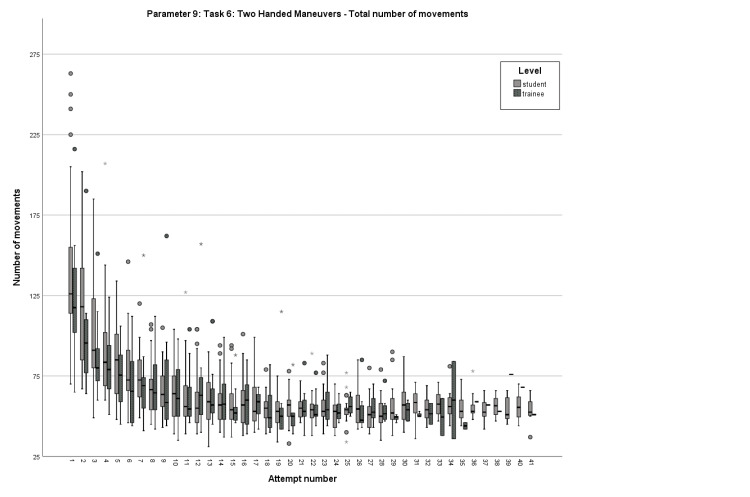
Box plot comparison of the learning curves regarding Parameter 9 Comparison of the total number of movements in Task 6 between students and residents for each attempt.

Concerning Parameter 9, the median number of movements on the first attempt for the students was 126, reaching a first pass on their ninth attempt and procuring a definitive pass on their 12^th^ attempt. On the same parameter, the median value for residents on their first attempt was 117.5 movements, with the first pass achieved on the ninth attempt and the definitive pass on the 14^th^ attempt (Figure 3).

**Figure 4:**
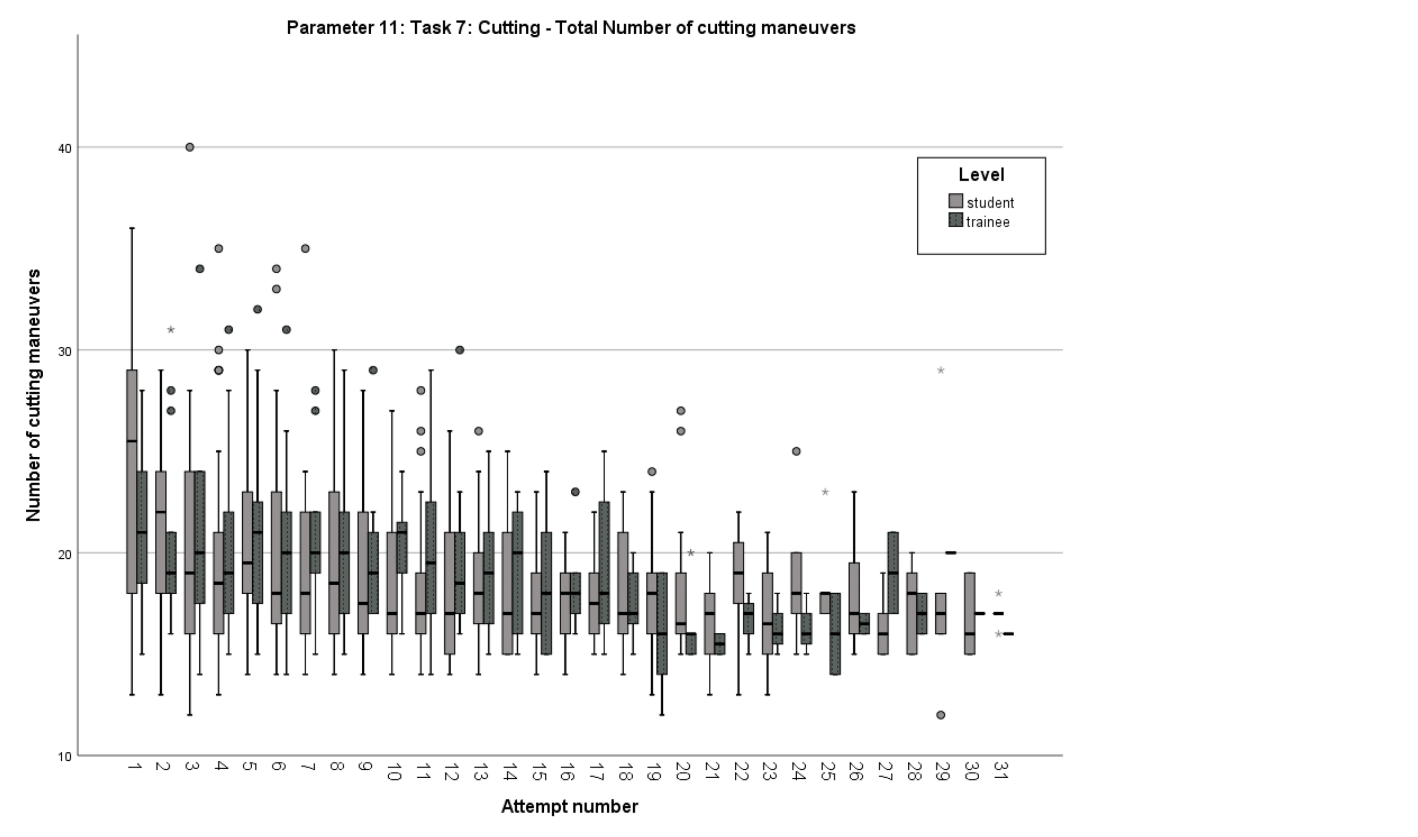
Box plot comparison of the learning curves regarding Parameter 11 Comparison of the total number of cutting maneuvers in Task 7 between students and residents for each attempt.

Lastly, on Parameter 11, students had a median starting performance of 25.5 cutting maneuvers, with the first pass recorded on the second attempt and the definitive pass on the fourth attempt. Residents commenced the task with a median rating of 21 cutting maneuvers, with a first pass detected on the second attempt and the definitive pass on the fifth attempt (Figure 4).

None of the above differences reached a statistically significant level. 

After examining the learning curves, certain curves exhibited critical points, whereas others showed a steady progression. For both groups, Parameter 1 displayed a curve progression that lacked statistical significance at any specific step (Figure 1). In contrast, Parameter 6 demonstrated statistical significance in the group of medical students when comparing their first and second attempts, with 185 movements on the first try and 142 movements on the second try (p=0.012) (Figure 2). In the same parameter and attempt numbers, the group of residents failed to reach statistical significance with a borderline p-value (p=0.06) and median values of 150 and 123.5, respectively. In Parameter 9, residents achieved statistical significance between their first and second attempts (p=0.021), with the corresponding attempt values being 117.5 and 95.5 movements (Figure 3). 

Statistical analysis was carried out to compare the two groups' first pass and definitive pass for each parameter. No significant difference was observed between students and residents in any parameter (Table 2). Overall curriculum's first pass and definitive pass were also compared, and no difference was shown between the two groups. The students' first pass of the curriculum was at the 27^th^ attempt, while residents accomplished this at the 25^th^ attempt (p=0.737). Definitive pass of the curriculum was achieved on the 28^th^ attempt for both groups (p=0.924). 

**Table 2:** Number of attempts at which the first pass and definitive pass were achieved across each studied parameter (P1-P13) between students (S) and residents (R).

First pass													
	P1	P2	P3	P4	P5	P6	P7	P8	P9	P10	P11	P12	P13
S	1	1	1	1	2	9	1	2	9	2	2	1	1
R	1	1	1	1	1	7	1	1	9	1	2	1	1
p	0.113	0.220	0.206	0.291	0.267	0.884	0.071	0.516	0.830	0.252	0.402	0.975	0.884
Definitive pass													
	P1	P2	P3	P4	P5	P6	P7	P8	P9	P10	P11	P12	P13
S	2	2	2	2	4	14	2	6	12	4	4	2	3
R	2	2	2	2	2	12	2	4	14	3	5	2	3
p	0.267	0.141	0.146	0.206	0.516	0.599	0.071	0.227	0.751	0.737	0.633	0.750	0.229

The primary objective of this study was to investigate possible differences in curriculum completion between medical students and surgical residents based on attempt number. The secondary aim was to ascertain any differences in the number of attempts made by the two groups on their first pass of the curriculum. However, the subsequent post-hoc analysis indicated that the study lacked sufficient power to draw conclusions. Group sizes were defined as 34 and 15, respectively. The resulting power was 0.15 for the primary objective and 0.22 for the secondary objective. The authors went on to determine the ideal study size. The allocation ratio was defined as 2:1. The ideal study would require 195 medical students and 98 surgical residents to achieve a power of 0.8 for the primary objective. 

## Discussion

Both students and residents were found to have statistically significantly different results in a few comparisons concerning specific task parameters and attempt numbers, but these differences did not affect the overall completion rate of the curriculum between the groups. Previous studies that aimed to develop an evidence-based curriculum had participants ranging from 33 to 58 [12-14] participants, while this study recruited 49 trainees. However, according to the post-hoc analysis, a larger sample size of 293 participants is necessary to reliably assess differences in the completion rate of the curriculum. 

No similar comparisons were identified in the literature on the rate at which different groups complete a predefined VR laparoscopic curriculum. Previous studies have focused mainly on differences in learning curves to justify the creation of a curriculum. Most studies have shown that novice surgeons can achieve expert proficiency in basic skills after approximately ten attempts, with individual variation [13]. This finding is consistent with this study for the most demanding tasks, in which the number of movements was one of the assessed metrics. As noted above, students reached proficiency on Parameter 6 on their 14^th^ attempt and residents on their 12^th^ attempt. Similarly, for Parameter 9, students reached proficiency on the 12^th^ attempt and residents on the 14^th^ attempt. There is extensive literature comparing cohort data of residents and expert surgeons to construct curricula that will enhance the educational program during residency, as educational opportunities in the operating room are diminishing and patient safety is a top priority [15]. Studies focusing on residents and experts have found statistically significant differences between the groups regarding learning curves [8, 9], including the number of movement-related parameters [13,14]. However, the population of the present study differs from the aforementioned studies, as medical students are generally considered laparoscopy-naive. Studies comparing residents and medical students are limited, as most research has focused on comparing two groups of medical students: those trained traditionally in the operating room and those trained in VR [16]. Only two studies were found that compared different groups in terms of learning curves and included medical students as participants. The first study aimed to develop a hysterectomy curriculum and found a statistically significant difference between medical students and residents regarding learning curves [12]. However, the inclusion criteria for medical students in this study required previous participation in three or more laparoscopic hysterectomies. The second study found no statistically significant difference between students on clinical rotation and first- or second-year residents in the same tasks aimed at improving laparoscopic assisting skills [17], with inclusion criteria requiring no prior laparoscopic simulation training. 

This study had several limitations. Its validity may have been affected by its single-center design. Additionally, the absence of structured residency training in this European country may have contributed to the lack of differences between the groups. Furthermore, the number of trainees was insufficient, as determined by the post-hoc analysis. However, this is the largest study conducted for this specific comparison, according to the authors’ best knowledge, and highlights the need for multicenter studies. The curriculum for this study was based on tasks from other studies [8, 9], which included participants who differed from those in this study. It is possible that the tasks used in the present study did not properly evaluate the differences in the studied groups. However, there is a lack of data regarding medical students in the literature; hence, there was no better way to plan the present study. Moreover, the study did not assess the retention of acquired skills, which could confirm whether the curriculum used was equally beneficial for both groups and whether the tasks used, derived from other studies, were the optimal choice. 

Simulation training is an emerging educational modality in medicine. The implementation of VR technology in the field of surgery is still at an early stage [18], which affects its use in the education of medical students. Therefore, recruiting participants for comparison between students and residents can be challenging. To gather the appropriate number of participants, a multi-center design could be employed. However, if a study enrolls participants from various countries, and among these countries, some offer structured surgical training while others don’t, this may introduce a significant heterogeneity issue. Moreover, this discrepancy could affect the interpretation of results, as residents in non-structured training programs may be considered laparoscopy-naive. To address this issue, residents could be stratified based on whether they attend structured training programs or not. Additionally, stratification based on the year of training may be useful for each group, as residents may accumulate different experiences and education in structured versus unstructured environments. Assessing retention is essential to identify potential differences between the two groups that may result in distinct approaches after completing the curriculum. Additionally, comparing the basic laparoscopic skills of the two groups in the context of a non-structured residency training program may be inadequate. It may be more appropriate to evaluate the pace at which these two groups complete a curriculum in a simulation of more complex procedures, such as cholecystectomy. Additional and more complex steps can enhance and highlight potential differences between the two groups. 

## Conclusions

In the present study, the overall learning pace in basic laparoscopic skills, through a VR simulator, did not differ significantly between medical students and surgical trainees. However, this might have been due to a small sample size. A sample size of close to 300 participants is required to provide a definitive answer. If a lack of difference is indeed confirmed in future studies, a common curriculum in basic laparoscopic skills is suggested for both students and residents.

## Figures and Tables

**Figure 5:**
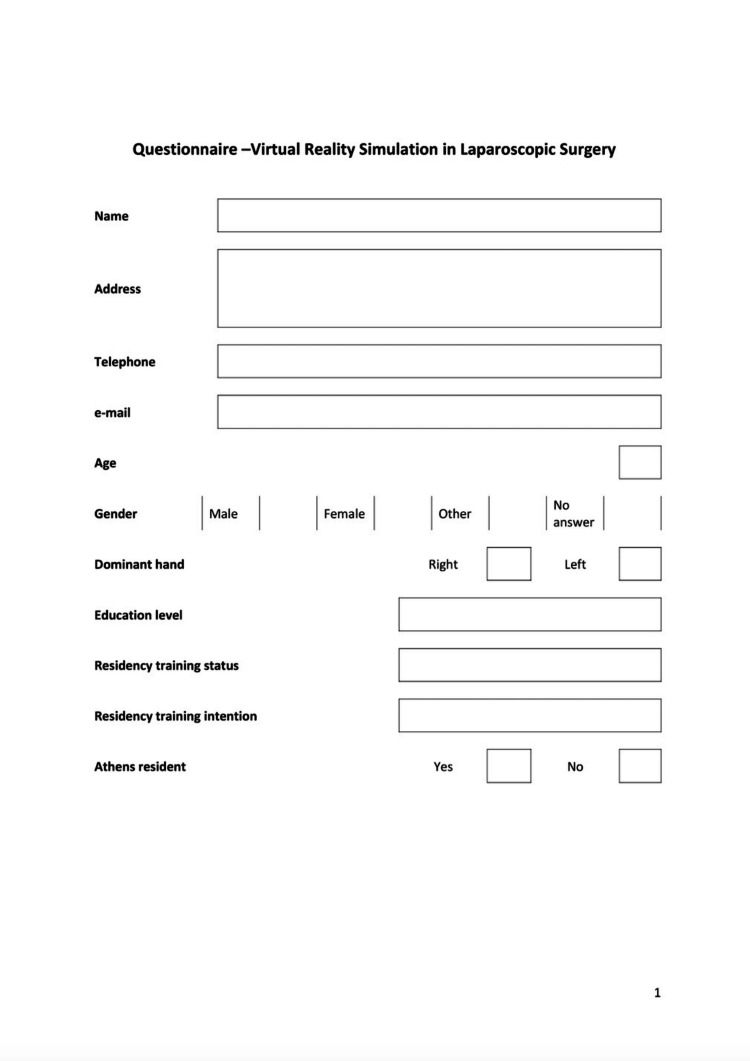
Questionnaire, Part 1 The questionnaire was distributed to participants upon beginning of the curriculum.

**Figure 6:**
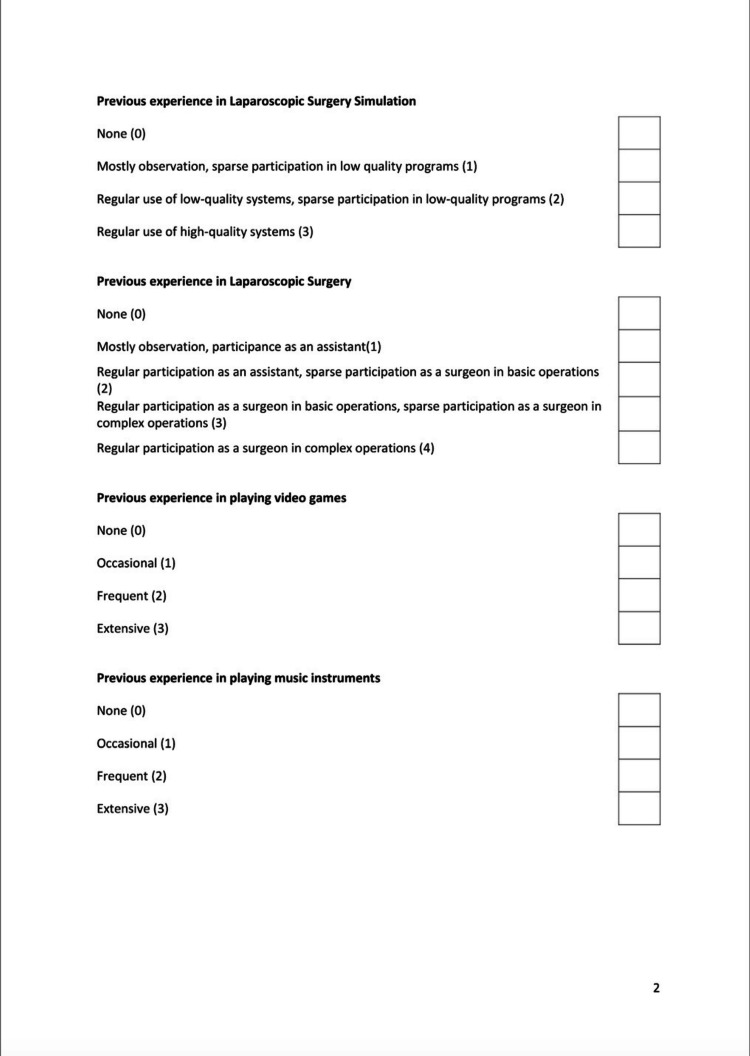
Questionnaire, Part 2 The questionnaire was distributed to participants upon beginning of the curriculum.

**Figure 7:**
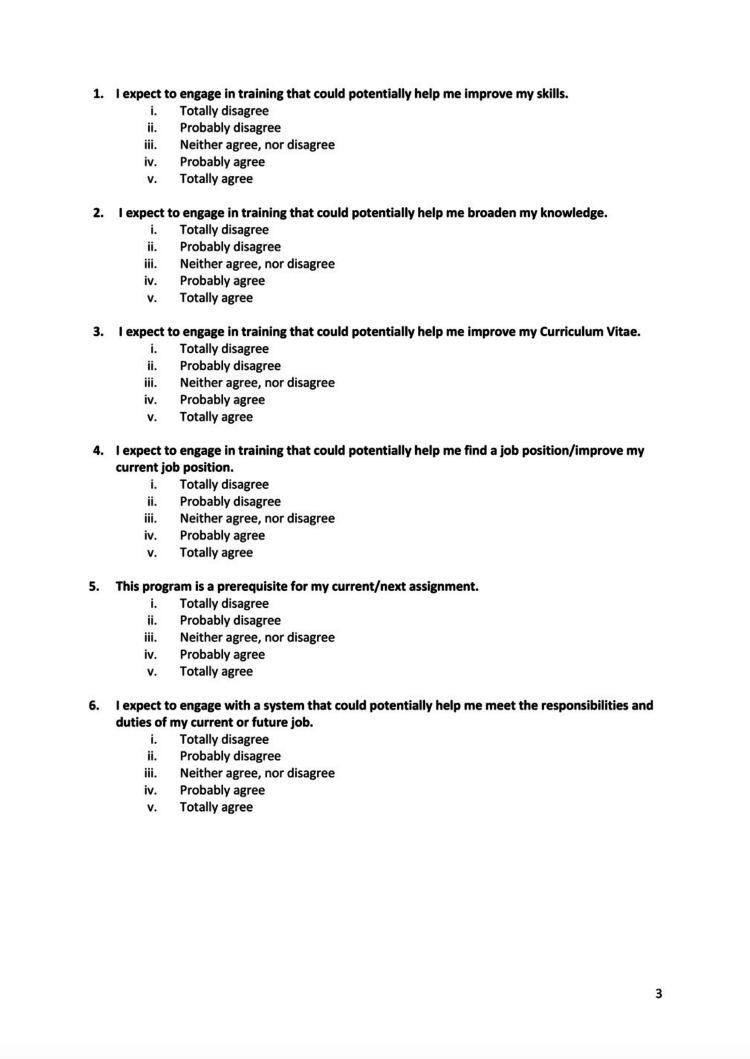
Questionnaire, Part 3 The questionnaire was distributed to participants upon beginning of the curriculum.

## Appendices

Appendix A 
